# Hepatic Artery Aneurysm—A Rare Cause of Cholestasis

**DOI:** 10.5334/jbsr.3751

**Published:** 2024-09-25

**Authors:** Sam Verrept, Wouter Van Den Eynde, Yves De Bruecker

**Affiliations:** 1 Radiology department Imelda Hospital, Bonheiden, Belgium; 2 Vascular surgery department Imelda Hospital, Bonheiden, Belgium; 3 Radiology department Imelda Hospital, Bonheiden, Belgium

**Keywords:** aneurysm, hepatic artery, cholestasis, endovascular stent, ERCP

## Abstract

Hepatic artery aneurysms (HAAs) are rare vascular malformations that can arise from atherosclerosis, trauma, or iatrogenic injury. HAAs can be symptomatic and lead to serious complications. We present the case of a patient with painless jaundice caused by obstruction of the distal common bile duct by a HAA on a replaced right hepatic artery. This was further complicated with cholangitis. After endovascular stenting of the aneurysm, cholestasis decreased.

*Learning point:* Hepatic artery aneurysms can cause common bile duct obstruction resulting in cholestasis.

## Case History

**Figure 1:**
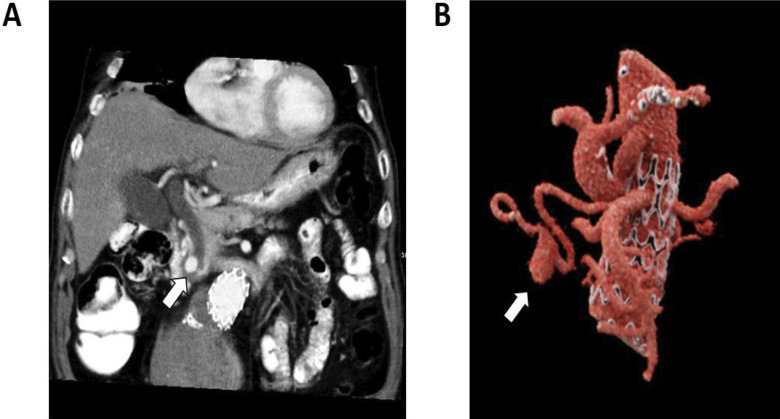
**(A)** Contrast-enhanced CT in an oblique coronal plan (MinIP recontruction) showing the right hepatic artery aneurysm at the level of the ampulla of Vater (arrow). Proximal dilation of the common bile duct is suggestive for obstruction. **(B)** VRT reconstruction showing a segment of the abdominal aorta with its branches: the celiac trunk, the superior mesenteric artery, from which the hepatic artery arises with the aneurysm (arrow), and the renal arteries.

A 77-year-old male patient presented at the emergency department with painless jaundice and fever. Biochemistry showed a cholestatic liver set with elevated bilirubin, together with leukocytosis and a high C-reactive protein (CRP). Abdominal computed tomography (CT) showed dilation of extra- and intrahepatic bile ducts with obstruction of the distal part of common bile duct. An aneurysm of the replaced right hepatic artery—an anatomic variant that branches off the superior mesenteric artery, which was located at the level of the ampulla of Vater—was considered as the most likely obstructive cause (Figure 1).

**Figure 2:**
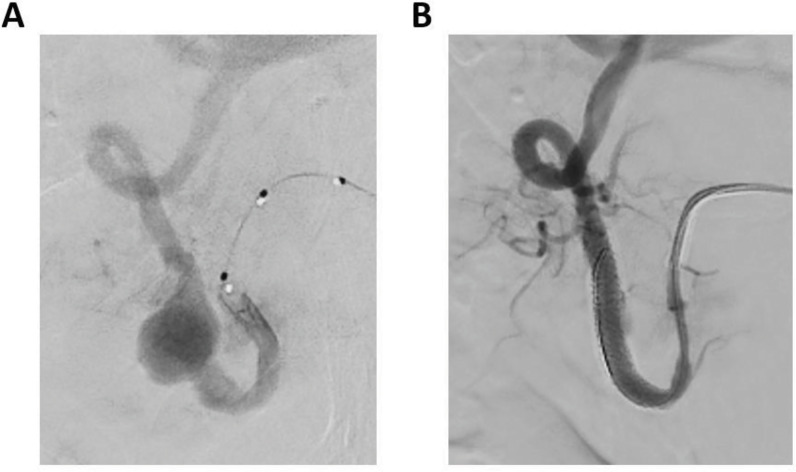
**(A)** Fluoroscopy images showing the endovascular catheterization of the hepatic artery and the aneurysm. **(B)** Situation after insertion of an expandable stent over the aneurysm, excluding it from the circulation.

A diagnosis of cholangitis with cholangiosepsis was made on the basis of hemocultures positive for *Escherichia coli*, for which intravenous (IV) antibiotics were initiated. An emergency endoscopic retrograde cholangiopancreatography (ERCP) procedure with papillotomy showed no bile stones, thus confirming the hepatic aneurysm as the cause of biliary obstruction. Subsequently, angiographic stenting of the right hepatic artery aneurysm was performed achieving exclusion of the aneurysm (Figure 2).

**Figure 3:**
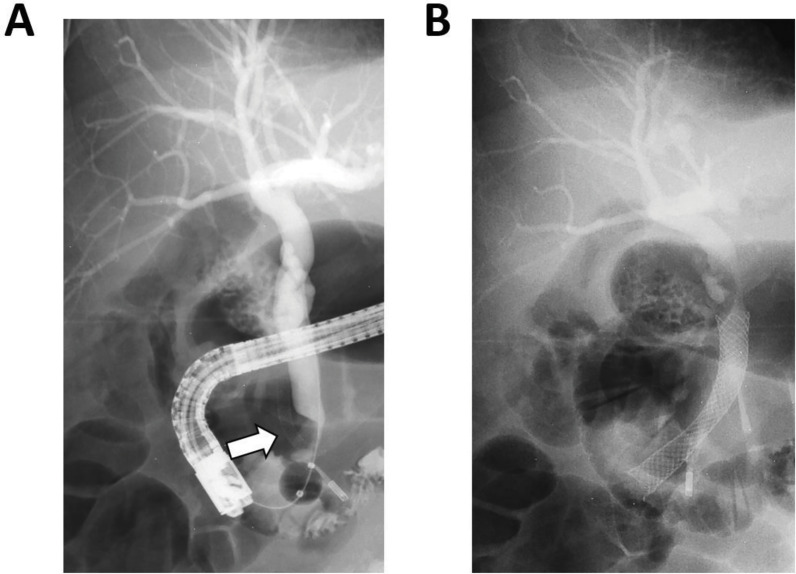
**(A)** Cholangiography images during the ERCP procedure showing dilated intra- and extrahepatic bile ducts with a persisting impression of the stented right hepatic artery aneurysm sac on the distal common bile duct (arrow). Multiple gallstones are in the gallbladder. **(B)** Situation after placement of a metal stent in the common bile duct.

A few days later the patient unfortunately developed a postpapillotomy bleeding with significant anemia. An urgent ERCP was performed, which showed blood clots in the common bile duct. On the accompanying cholangiogram, the external compression by the stented right hepatic artery aneurysm is still visible, however, without complete obstruction of the common bile duct. For hemostatic purposes, a metal stent was placed in the common bile duct (Figure 3).

**Figure 4:**
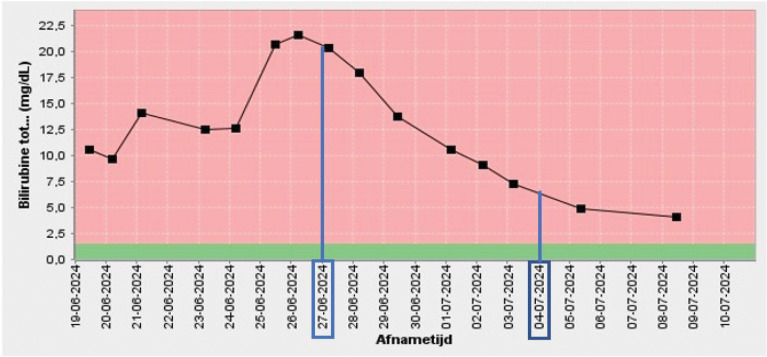
Graph showing the evolution of the total bilirubin plasma level reflecting cholestasis in function of time. After the aneurysm stenting on 27 June 2024, an evident decrease in cholestasis is noted. This decrease levels out after placement of the metal stent in the common bile duct on 4 July 2024.

During further observation, there was a favorable clinical and biochemical evolution. The latter is demonstrated by a significant decrease in the total bilirubin plasma levels from the moment of stenting of the hepatic aneurysm (Figure 4).

## Comments

Hepatic artery aneurysms (HAAs) are part of the group of visceral artery aneurysms (VAAs)—aneurysms affecting the celiac or mesenteric arteries and their branches. These are relatively rare, with a reported incidence less than 0.2%, which might be an underestimation since many remain asymptomatic [1]. True aneurysms involve all layers of the wall, while pseudoaneurysms result from a tear in the vessel wall (due to trauma) with subsequent periarterial hematoma formation. The advance of percutaneous biliary procedures (e.g., ERCPs) has probably increased the incidence of iatrogenic hepatic artery pseudoaneurysms, making the hepatic artery the most commonly reported site of VAA. However, atherosclerosis is still considered the most important etiology of HAAs. The majority of hepatic artery aneurysms are extrahepatic [2].

Symptomatic HAAs can present with epigastric pain [3]. Complications of untreated HAAs include biliary obstruction (resulting in cholestasis) [4], erosion into the biliary tree (resulting in haemobilia) [5], erosion into the portal vein (resulting in portal hypertension) [6], or rupture into the retroperitoneal space and/or peritoneal cavity. A retrospective case series reported a 14% incidence of rupture of HAAs [7].

The general approach to VAAs is early elective treatment, rather than watchful waiting, to minimize the risk of complications. This especially accounts for pseudoaneurysms, which have a high risk of rupture. In the elective setting, endovascular treatment is preferred above open surgical repair because of its lower morbidity and mortality rates [2].

In this case, the HAA was located on a replaced right hepatic artery (arising from the superior mesenteric artery (SMA) instead of the celiac trunk). This variant in vascular anatomy is present in ~4% of the population [8]. It is probably due to the relatively lower course of the right hepatic artery that the HAA was able to compress the common bile duct at the level of its outlet in the ampulla of Vater. Because of the absence of previous biliary procedures or significant trauma, atherosclerosis is the most likely cause of the aneurysm in this high-risk cardiovascular patient. This case illustrates that such a HAA can potentially cause bile duct obstruction with cholestasis that can be relieved by endovascular treatment.
